# Cracking the egg: the use of modern and fossil eggs for ecological, environmental and biological interpretation

**DOI:** 10.1098/rsos.180006

**Published:** 2018-06-20

**Authors:** Shaena Montanari

**Affiliations:** School of GeoSciences, University of Edinburgh, Edinburgh EH9 3FE, UK

**Keywords:** eggs, palaeoecology, palaeobiology, ecology, environmental reconstruction

## Abstract

A myriad of extant and extinct vertebrates produce eggs. Eggs and eggshells provide a useful substrate for reconstructing environment, ecology and biology over a range of time scales from deep time to the present. In this review, methods for analysing and understanding records of diet, climate, environment and biology preserved in eggshells are presented. Topics covered include eggshell structure, assessing diagenesis, stable isotope geochemistry and morphological investigations of eggshell characteristics. This review emphasizes the use of eggshells in the modern and fossil record, as they allow for interpretation of characteristics of a wide variety of amniotes across geological history, uniquely informing environmental and ecological investigations.

## 1. Introduction

**Figure 1.**
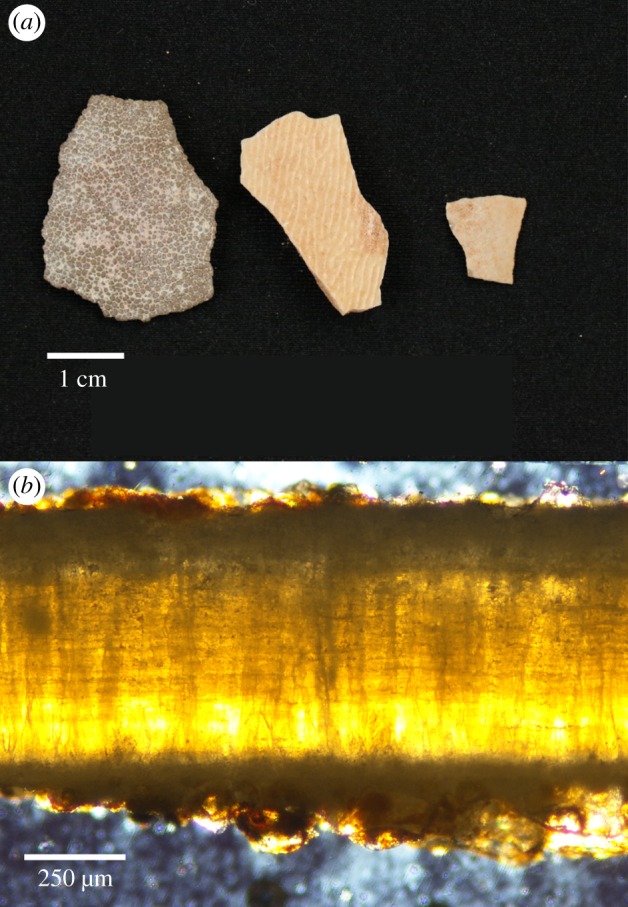
Examples of fossil eggshell. (*a*) Three examples of non-avian dinosaur eggshell fragments from Mongolia's Gobi Desert that are often found as remnants in the fossil record. Different eggshell types possess varied forms of ornamentation and can generally be attributed to different groups of non-avian dinosaurs, like (from left to right) (putative) sauropod, oviraptorid and (putative) troodontid. (*b*) A radial thin section of a troodontid dinosaur eggshell viewed in transmitted light. The distinct inner mammillae layer and outer prismatic layer are visible.

**Figure 2.**
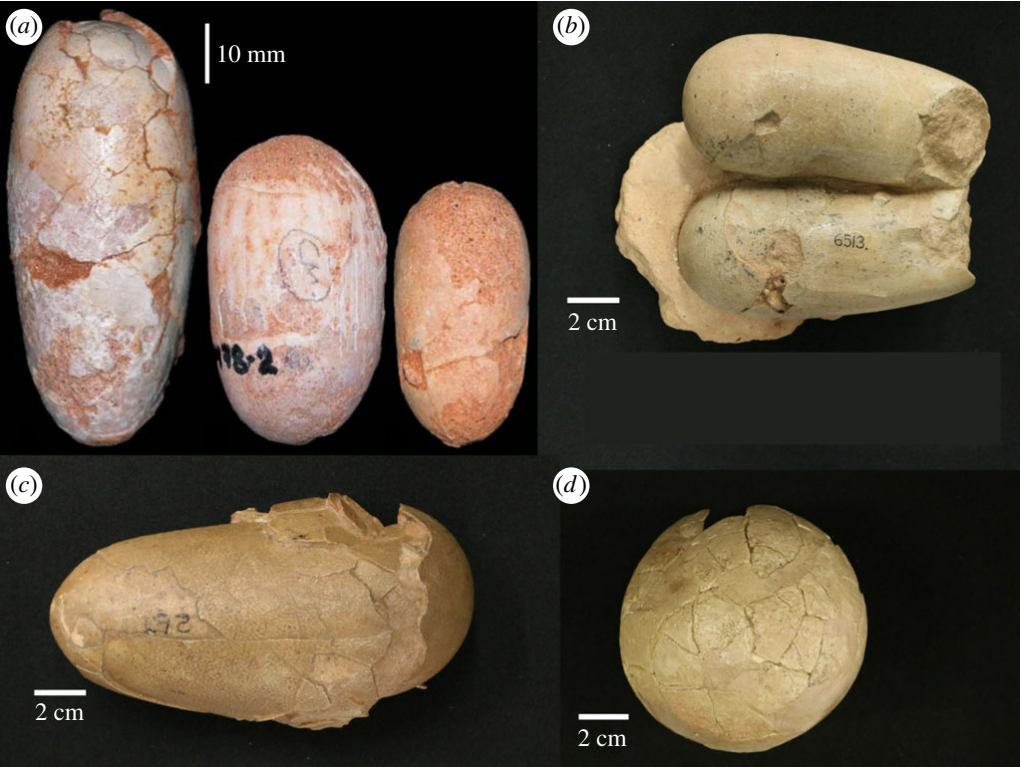
Examples of whole or partial fossilized eggs. (*a*) Examples of three avian eggs from Mongolia's Gobi Desert, from Varrichio & Barta [2]. (*b*) Unidentified theropod dinosaur eggs from the Cretaceous of Mongolia (AMNH FR 6513). (*c*) An oviraptorid dinosaur egg from the Cretaceous of Mongolia (AMNH FR 6508). (*d*) A probable ornithopod dinosaur egg from the Cretaceous of Mongolia (AMNH Field no. 707).

To find out information about animals, palaeontologists and biologists alike often look to bodies and bones. Often, other materials left behind in both modern environments and the fossil record are overlooked. Shelled eggs of reptiles, birds (including non-avian dinosaurs) and monotremes are laid on land and have been for over 200 million years [1]. Extinct non-avian dinosaur (hereafter referred to as dinosaurs) eggs were not much different than species of their extant relatives, as fossil eggs have been discovered that look remarkably similar to modern birds (figures 1 and 2). Eggs provide another biogenically created material that can be used to reveal specific information about the egg-layers and the environments they live in when assessed with different types of geochemical, morphological and molecular techniques.

Eggshells are found in modern environments, as remains in archaeological and palaeontological sites, and preserved in well-catalogued historical museum collections. In modern settings, hatched eggs can be used for study, providing a non-invasive way to study certain species without handling live animals. These invaluable records allow us to trace changes in biology, environment and ecology over time [3]. Although not covered in this review, eggshells can even provide a record of environmental exposure to mercury [4,5]. Eggshells are a suitable substrate for many types of invasive analysis because they are often fragmented and sampling does not require destruction of a large amount of material, which causes only small amounts of damage to collections if the eggshell specimens are limited. Outside of modern eggshell finds, it is often possible to identify eggshells to a species level when found in an archaeological site, and even sometimes in palaeontological sites, making inferences even more powerful. This is also true with dinosaurs, as there are instances where eggs and adult dinosaur remains can be identified and associated [6]. Ostrich eggshell is frequently found at archaeological sites as food waste, bead decorations or water carriers in northern China, Mongolia [7], southern Africa [8], northern Africa [9] and India [10]. This can provide direct evidence of what environments were like at the time of human occupation in localities where we would not normally be able to glean this information.

Eggshells have both an organic and inorganic portion that can be preserved in the environment for thousands (organic) to millions (inorganic) of years and can be directly dated using a variety of methods [11], increasing their use as an information archive. In this review, I will explore how the structure of both modern and fossil eggshells allows them to be used as ecological and environmental proxies for local vegetation, hydrology, aridity and past levels of atmospheric O_2_. In addition to ecology and environment, eggshells are strong biological indicators, as the organic portion of the shell can contain both modern and ancient DNA (aDNA). Measuring the pores in eggshells reveals what types of nests they were buried in, hinting at the evolution of nesting behaviour over millions of years. Advanced geochemical techniques allow for the measurement of body temperatures from eggshell crystals, opening up new doors for understanding palaeobiology and palaeoecology.

## 2. Eggshell structure

### 2.1. Mineral and organic structure of eggshells

**Figure 3.**
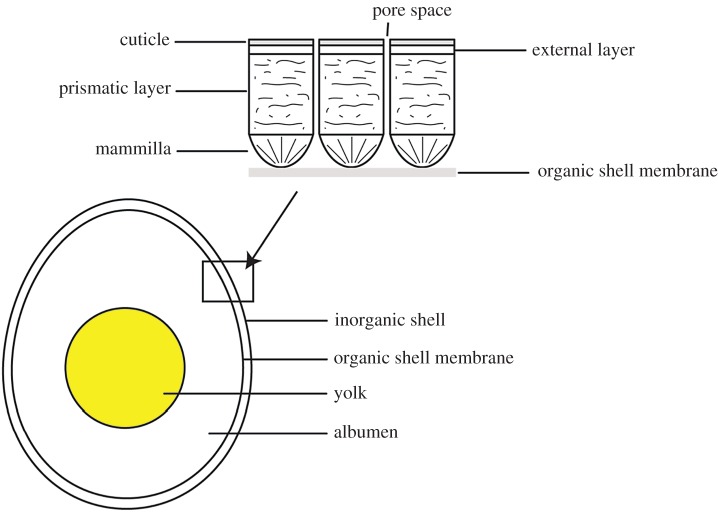
A general schematic of a hard-shelled vertebrate egg. The vertebrate egg consists of a yolk, surrounded by albumen (egg white). This is contained in an organic shell membrane all held in by a crystalline calcite shell on the outside made up of a three distinct layers. The inner surface of the calcite shell has radiating cones (mammillae) that are anchored at their tip to an organic membrane that encases the yolk and albumen. The prismatic layer and external layer make up the outer part of the eggshell.

Both reptile and avian eggs have essentially similar structures. A basic vertebrate egg consists of a yolk, surrounded by albumen (egg white), which is contained in a shell membrane all held in by a crystalline shell on the outside (figure 3). Bird eggshells are composed of columnar calcite (CaCO_3_) crystals with an organic matrix of protein fibres throughout. The inner surface of the calcite shell has radiating cones (mammillae) that are anchored at their tip to an organic membrane that encases the yolk and albumen. Eggshell pores are present along calcite crystal boundaries to provide a mechanism for gas exchange while the eggs are incubating [12]. Most extinct non-avian theropod eggs that have been discovered only have two eggshell layers, lacking the external layer that is seen in crown group birds. There is a transparent cuticle on the outside of the shell that protects matter from entering the pores. In general, a bird egg is approximately 95% inorganic matter, 3.5% organic matter and 1.5% water [12]. The structure of eggshell lends itself well to preservation in deep time. The intracrystalline organic matrix is preserved relatively well in a closed system, even at high temperatures, leading to its suitability for DNA and amino acid analysis [13]. Eggshell retains a much higher percentage of its indigenous amino acid composition compared to bone under the same experimental diagenesis conditions [14], making it more favourable to use in analyses where organic chemistry or aDNA analysis is desired.

Hirsch [15] describes in detail the structure of chelonian (turtle) eggshell in comparison to avian, dinosaurian and other reptilian eggshell. Chelonian eggshells are the only eggshells composed of aragonite as opposed to calcite. Hirsch [15] also noted that there are three ‘types’ of eggshells, soft, pliable and rigid shelled, and that chelonians have both pliable and rigid-shelled eggs. Soft-shelled eggs seen in snakes, lizards and the tuatara have a shell made primarily of organic membrane, with no organized calcareous layer. There are only unorganized, small floating crystals or a calcareous ‘crust’ on the outside of the egg. This egg type is very unlikely to fossilize, although there are instances of exceptionally preserved eggs of this type from a Cretaceous choristoderan reptile [16] and an unusually large cache of pterosaur eggs [17].

Pliable-shelled eggs are seen in sea turtles and other types of turtles. While their shells are still pliable, they have a thicker, more organized calcareous shell than other soft-shelled eggs [15]. Compared to other sorts of turtles with this eggshell type, sea turtles have the thinnest calcareous layer, and their chances of fossilization are poor. Rigid-shelled eggs are most commonly preserved in the fossil record owing to their thick calcareous layers composed of interlocking crystals that will not easily dissociate. Tortoises, some geckos, all crocodiles, birds and dinosaurs have this type of eggshell. When compared to soft-shelled eggs, rigid-shelled eggs have a far smaller organic component, reduced only to a thin membrane inside the shell and a delicate network of collagen fibres in the calcareous layers [15]. Currently, there is a lack of research on the detailed structure of squamate and monotreme eggs in both the modern and fossil record, so the specifics about their eggshell construction are not known.

### 2.2. Assessing diagenesis and alteration

Diagenesis in eggshells has primarily been studied in fossil aves and non-avian dinosaurs. While it appears that the organic portion of eggshell typically only persists for thousands of years [18], the inorganic portion, if it is not severely altered, may contain useful palaeoenvironmental and palaeobiological information for tens of millions of years. Diagenesis can alter the physical crystal structure and/or the chemical signature in calcium carbonate eggshell. If a fossil is composed of aragonite, it will commonly alter to calcite; if it is originally calcite, as in bird, dinosaur and reptile eggshells, the status of alteration may be more difficult to assess. X-ray diffraction is commonly used to visualize alteration of aragonite to calcite, i.e. in corals [19], but this will not help distinguish between primary and secondary calcite alteration. When the study focus is stable isotope analysis, a main method of assessing for diagenesis is isotopic comparisons of material at a site. If the measured isotope ratios of abiotically derived soil carbonates, bone, and other materials are significantly different, this is thought to indicate that overwhelming alteration has not impacted the isotopic ratios of all materials at the site [20]. Since there is no one way to determine the level of diagenesis, it is better to use a variety of methods to assess potential alteration in eggshells and other materials. A suite of methods must be used to assess alteration and determine if fossil eggshell remains are suitable for either stable isotope or DNA analysis.

Cathodoluminescence (CL) is a method for assessing diagenesis that has commonly been used to examine patterns of alteration in calcite shells [21]. It is a method employed using a CL detector attached to a scanning electron microscope (SEM). The CL detector measures the wavelengths of the photons being emitted from the mineral while it is being bombarded with high energy electrons in the SEM. Commonly, manganese (Mn) and iron (Fe) will replace calcium (Ca) in a crystal lattice [22]. There is bright fluorescence in CaCO_3_ if there is substitution of Mn^2+^ in the crystal lattice in place of Ca; the presence of Fe^2+^ will quench luminescence [23]. Unaltered CaCO_3_ only fluoresces lightly under CL, and the biogenic calcite of eggshells does not contain Mn and Fe naturally, so CL can be used to determine alteration in these fossils [22]. This method can also help visualize where alteration is, determining if it pervasive throughout the shell or if it is localized to pores or outer surfaces of the shell. Epifluorescent light microscopy has also been used as an alternative to CL because it does not involve as much sample preparation and, therefore, saves time and money in fossil eggshell analysis [24]. Unlike CL, it can be used to analyse the amount of organic matter in carbonates, and could provide yet another viable method of assessing eggshell alteration [24].

Transmitted light microscopy (TLM) using a petrographic microscope and SEM also provides clues about the alteration of fossil eggshells. Examining a thin section of an eggshell through TLM gives a strong indication of the preservation of original crystal eggshell structures and layers. Intrusive pore waters can dissolve crystals of eggshells after burial, and these waters can also imprint a different chemical signature on the eggshells if they are dissolved. Not being able to identify specific layers such as the mammillae may indicate the eggshell has been recrystallized. SEM analysis can also help visualize crystal structure and layers of eggshell in order to assess alteration. While using the SEM, different modes such as electron backscatter diffraction (EBSD) and energy-dispersive X-ray spectroscopy (EDS) can be used to understand in greater detail specific zones of alteration and their elemental composition [25].

As previously mentioned, iron will quench luminescence, so EBSD can be used to determine where in the fossil iron might be present if it does not luminesce under CL. The backscatter (BSE) detector in an SEM measures the number of electrons that reach it, and this number is inversely proportional to the size of the atom the electrons collided with; atoms with a low atomic number will show up as a dark area in the BSE image, while atoms with a high atomic number will show up lighter in the BSE image. EBSD has been used to study crystallographic patterns in avian eggshell in order to understand mineralization and behaviour of trace elements [26]. This method is often used in conjunction with EDS integrated into the SEM to fine-tune elemental maps of a specimen. The EDS X-ray detector detects characteristic elemental X-rays and the software then analyses the resulting energy spectrum to determine the types and abundances of elements. This technique can be useful when creating an elemental map of a fossil specimen to determine zones of alteration, and is helpful in identifying elements that are not typically present in biogenic calcite, which will indicate alteration has taken place. EDS has shown to be effective for examining mineral and elemental alteration in fossil dinosaur eggshells [20,22].

In addition to the above-mentioned methods, other methods such as electron microprobe and X-ray absorption near-edge spectroscopy analyses have been used to look at eggshell alteration in birds with good success [27]. Grellet-Tinner *et al.* [20] provides a useful flowchart for determining the best combination of methods to use in assessing diagenesis and alteration in fossil eggshells. They note that most studies only use one of these methods to determine if alteration has taken place, when owing to the strengths and weaknesses of each method, a combination of two or more visualization and microscopy techniques would be most effective. In the supplemental material of Eagle *et al.* [28] a similar multi-step method is used to determine if the eggshell structure has been both chemically and physically altered. If the combined evidence shows the eggshell has most likely not been seriously altered (using a suite of methods mentioned in this section), it is deemed appropriate to use it for stable isotope analysis.

## 3. Ecological and environmental indicators

### 3.1. Stable isotope ecology

In the past decade, using stable isotope analysis on eggshells and other egg products to estimate environmental and ecological characteristics has proved to be a useful tool for both modern and fossil analyses. It is well established that stable isotopes of carbon (*δ*^13^C), nitrogen (*δ*^15^N) and oxygen (*δ*^18^O) of biogenic materials such as teeth, bone, collagen, feathers, hair and eggshells can yield information about ecology, diets, migration patterns and behaviour of animals [29,30]. Eggshell and other egg products have been relatively underused in stable isotope research, but possess many useful qualities that indicate they can and should be exploited for geochemical analysis.

Pioneering work in the late 1970s through to the 1980s established a firm foundation for understanding how the stable isotopes of nitrogen, carbon and oxygen contained in animal diets were reflected in the tissues of an individual [31–33]. This research laid the groundwork for our understanding of how stable isotopes interact with live organisms both in modern ecology and palaeoecology. Carbon and nitrogen isotopes reflect the diet of the animals, while oxygen isotopes in animal tissues are primarily sourced from ingested environmental water. Only a short review on stable isotopes in biology and ecology will be provided here, for a more detailed review, see Koch [30,34].

The *δ*^13^C ratio found in the inorganic and organic portions of the egg is relative to the *δ*^13^C of ingested organic material [34]. In herbivores, this is ingested plant material, but in omnivores and carnivores their *δ*^13^C value also carries the *δ*^13^C signal of their prey, and in turn the primary producers at the bottom of the food web. The different photosynthetic pathways, C3 (Calvin-Benson) and C4 (Hatch-Slack), are characterized by different *δ*^13^C values. This is reflected in the *δ*^13^C value of the egg materials. C3 plants have a *δ*^13^C ranging from −32‰ in understory canopy conditions to −21‰ in drier environments [35]. Generally, the *δ*^13^C of C3 plants increases as the climate gets drier. C4 plants, which include grasses and food crops, range from −21 to −9‰. C3 plants dominate in cool, moist regimes.

*δ*^15^N can be measured in organic portions of animal remains, and in the case of eggs can be analysed in the yolk, albumen, membrane and organic shell matrix. The nitrogen in animal protein is almost entirely composed from nitrogen in the diet of the organism. The enrichment of *δ*^15^N between diet and organic tissue in vertebrates is generally thought to be between 3 and 5‰ [32]. The basis of the nitrogen cycle for consumption by egg-laying vertebrates is in plants; plants take up nitrogen from the soil, and this *δ*^15^N signature will vary geographically. There is a marked negative correlation between rainfall abundance and plant *δ*^15^N [36], which can be useful for reconstructing environments using eggshell samples. Trophic level can also be traced using *δ*^15^N. Each consumer's *δ*^15^N will become enriched relative to the food they consume, which continues up the food web with isotope ratios becoming enriched 3–4‰ at each trophic level [37].

Oxygen isotopes in water vary due to temperature, evaporation and source of air masses [38]. Terrestrial vertebrates do not directly ingest precipitation; instead, their water is primarily ingested from streams, ponds, lakes and leaves. These reservoirs typically have different *δ*^18^O than precipitation due to preferential incorporation of the ^18^O isotope into condensate during evaporation. The *δ*^18^O of an organism's body water is composed mainly of drinking water and can be used to reconstruct the landscape hydrology in palaeoenvironments. Organisms that obtain most of their body water from evaporatively enriched leaf water will have high *δ*^18^O values that differ more than expected from meteoric water values. Oxygen isotopes can be a useful tool in determining habitat, terrestrial land use, diet and physiology in little known extant and extinct species [30].

### 3.2. Captive and laboratory tests

The first published experiment on stable isotopic systematics in eggshells was in Folinsbee *et al.* [39]. They completed controlled diet and water experiments with chickens to investigate the timing of diet turnover in both organic and inorganic portions of the eggshell. They found a linear relationship between *δ*^18^O in water and inorganic eggshell carbonate, and saw this as a useful proxy for reconstructing palaeoenvironments. Later, Johnson *et al.* [18] would find that the linear relationship between *δ*^18^O of eggshell carbonate and water source did not hold true in wild ostriches, indicating that this relationship might not be a reasonable assumption to make in open ecosystems where body water is obtained from a variety of sources such as plants. Lazzerini *et al.* [40] calculated the oxygen isotope fractionation between ingested water and eggshell calcite and found it is in fact correlated in numerous bird species and could be used for palaeoenvironmental reconstruction, but the amount of enrichment was different for terrestrial and semi-aquatic birds. Even if absolute oxygen isotope fractionation factors are not known, Miller & Fogel [41] illustrated with modern emu eggshell from around Australia that eggshell *δ*^18^O can be a strong proxy for both modern and palaeo-aridity, emphasizing the importance of *δ*^18^O in the inorganic portion of eggshells for reconstructing palaeoenvironments.

The first paper explicitly exploring the sources and variation of carbon isotopes in eggshells was von Schirnding *et al.* [12]. They examined captive ostriches in South Africa to understand relationships between diet and *δ*^13^C of the inorganic and organic portion of the eggshell. At the end of their captive experiment it was shown that ostriches in different environments with different food sources had a remarkably consistent *δ*^13^C diet to tissue fractionation of 2.1‰ (organic portion) and 16.2‰ (inorganic portion), meaning eggs could be a reliable substrate for stable isotope analysis. Schaffner & Swart [42] conducted a similar study to understand fractionation between diet and tissue in eggs, but using seven species of wild seabirds: Caspian tern (*Sterna caspia*), elegant tern (*Sterna elegans*), laughing gulls (*Larus atricilla*), sooty tern (*Sterna fuscata*), white tailed tropicbird (*Phaethon lepturus*) and red billed tropicbird (*Phaethon aethereus*). They collected the majority of their samples from spent or broken eggs, which reduced the need for invasive methods of dietary study. They found the enrichment between diet and the inorganic portion of the eggshell was between 14 and 15‰ instead of the previously recorded 16.2‰ [12]. What was evident was a gradient between freshwater, estuarine, offshore and pelagic species in both oxygen and carbon isotopes. They concluded that remains of eggshells would be a reliable, non-invasive substrate for determining diet in environments where continuous invasive collection would not be possible.

There are not many captive experiments that definitively determine the sources of isotopic variation in each egg material (e.g. yolk, organic membrane, inorganic component) except for some recent research [40,43]. In Hobson [44] an experiment on multiple bird species using a variety of diets with different isotopic values showed that albumen, shell carbonate and shell membrane *δ*^13^C values indicate a diet integrated over 3–5 days, while the yolk is closer to 8 days. Knowing the time to integration of diet to biogenic material is important for looking at diet and environment in extinct birds. Using gentoo penguins, Polito *et al.* [43] were able to illustrate the different discrimination factors between diet (whole fish) and each portion of the egg: eggshell organics, carbonates, shell membrane, albumen and yolk. The results showed that the discrimination factor of *δ*^13^C in the inorganic carbonate of the eggshell was far lower than in previously examined birds. Gentoo penguins have a discrimination factor of approximately 7‰ between diet and eggshell carbonate, while discrimination between diet and shell membrane is 2.9‰, which is concordant with values from Hobson [44]. The *δ*^13^C and *δ*^15^N enrichment in yolk is 0.0 and 3.5‰, respectively, and in albumen it is 0.8 and 4.7‰. They showed that it is possible to reconstruct diet of these penguins when the right discrimination factors are taken into account; without their experiments, estimations of past environments and ecologies of these penguins would have been grossly misinformed. This work on the isotope systematics has laid the groundwork for other studies that use modern eggshells and egg membranes to make ecological, environmental and dietary inferences in water birds [45,46], chickens [47] and penguins [48], illustrating the use of eggs for modern ecology and conservation.

Most studies of fossil and modern eggs using stable isotope analysis are predominantly about birds, but there are a handful of studies that have used reptile eggs. Burleigh & Arnold [49] looked at a small number of island tortoise eggshells to understand niche partitioning between extinct species. A few papers that have mainly focused on dinosaur eggshells have also included some samples of turtle and/or crocodile eggs [50,51]. Beyond that, there has been no stable isotope research done on reptile eggshells or captive feeding experiments to test diet–tissue discrimination factors.

### 3.3. Fossil eggshell studies

After the work of Folinsbee *et al.* [39] on stable isotopes in eggshell, dinosaur palaeontologists understood the potential use of this tool for determining Mesozoic palaeoenvironments. The first paper specifically focused on dinosaur eggshell chemistry was that of Erben *et al.* [50], who attributed the decline of dinosaur species to pathologies in their eggshells. They included dinosaur shells from fossil sites in Utah, Spain, France and Mongolia, along with comparisons to a variety of bird and reptile shells to verify the conclusion drawn in Folinsbee *et al.* [39]. They purportedly identified eggs with specific dinosaur species, but this was merely conjecture and weakened some of their assumptions. Sarkar *et al.* [51] showed that stable isotope ratios in eggshell carbonate from the Lameta beds of India reflected a semi-arid environment with a definite C3-plant diet signal and evaporated pools for drinking water.

More detailed dinosaur eggshell stable isotope research followed with the work of Cojan *et al.* [52] on *δ*^13^C and *δ*^18^O in eggshell carbonate shifts throughout a stratigraphic section in France. They were able to determine, owing to accurate stratigraphic sampling, that the dinosaur eggshells showed a change in evaporation over time when compared with associated soil carbonates. Zhao & Yan [53] were able to sample across the Cretaceous–Palaeogene transition in China; they noted a trend towards more negative values in *δ*^13^C, and therefore diet, towards the boundary. They also found additional information in the eggshells, such as pathologies and *δ*^18^O anomalies, which they suggested indicated stress was being put on the dinosaurs from the environment [53]. In Central Asia, dinosaur eggshells from Gobi Desert Late Cretaceous localities have been analysed and indicate clear variation in the palaeoenvironments within one region during the same time period [20]. Dinosaur eggshells from the Late Cretaceous of Romania indicated the environment there had humid microhabitats where the dinosaurs were laying their eggs [54]. Riera *et al.* [55] looked at the dinosaur eggshells of the Southern Pyrenees in Spain and found while some eggshells could be useful for environmental reconstruction, comparison with carbonate nodules is necessary because diagenesis is prevalent.

In addition to understanding the relationship between modern ostrich diet and eggshell stable isotope values, Johnson *et al.* [18] illustrated fossil ratite eggshells could be a viable source of information for palaeoenvironmental reconstruction. Fossil ratite bird eggshells have proved most useful for these sorts of analyses owing to the fact they are robust and present in the fossil record from at least the Neogene to present. Emu eggshells were used to illustrate the strength of the Australian monsoon over the last 65 000 years based on the prevalence of C4 grass in their diet [56]. An archive of the change from a C3-dominated ecosystem to one with both C3 and C4 mosaic vegetation was discovered in ostrich eggshells of northern Pakistan and India [57]. The change in the composition of grasslands from the Early Miocene until the present was tracked using eggshells from species of both fossil and recent ratites [58]. Both *δ*^18^O and *δ*^13^C of extinct elephant bird *Aepyornis* indicate it ate primarily C3 vegetation in the Holocene landscape of Madagascar, and also had a *δ*^18^O less influenced by evaporation than modern ostriches [59]. These studies show the power of stable isotope analysis on ratite eggshells for tracking vegetation changes, which can reveal ecological shifts that could impact the survival of these species over time.

Newsome *et al.* [60] examined the *δ*^15^N in extant and fossil emu (*Dromaius novaehollandiae*) eggshells and fossil *Genyornis newtoni* eggshells over a 130 ka time scale to better understand the causes of megafauna extinction in the late Pleistocene of Australia. They found a significant rise in *δ*^15^N of the eggshell values from the Last Glacial Maximum to the Holocene, indicating more frequent arid conditions. The *δ*^13^C values decreased in *Dromaius* but not in *Genyornis* prior to the megafauna extinctions [60]. A high-resolution study of *Dromaius* eggshells exploiting the prevalence of these fossils around sites in Australia has shown that *δ*^18^O does not show an increase in aridity through 100 ka time sequences [61]. These studies indicate the extinction of these giant birds may have been human mediated, as no massive shift in climate is detected in their eggshell. Additional studies of ratite eggshells have been able to provide high-resolution climate records in South Africa [62] and France [63], illustrating a valuable use of bird eggshell that is common at many fossil and archaeological localities.

Besides ratite birds, penguin eggshells in Antarctica have been a popular substrate for isotopic analysis. Carbon isotopic composition of Adélie penguin (*Pygocelis adeliae*) eggshells illustrated a recent shift to lower trophic level prey in the last 200 years when compared with the previous 38 000 years [64]. Oxygen isotope records of Adélie penguin eggshells over a span of 8000 years reconstruct the palaeoenvironment of Terra Nova Bay, Antarctica [65]. Further work on penguins has helped elucidate the change in penguin diets as krill populations have waxed and waned [48]. Stable isotope analysis in eggshells is gaining popularity in Holocene records, as many eggs are preserved in cold environments and create a time series that is unique to studying ecological changes over thousands of years.

Besides traditional stable isotope methods, newer, more precise methods of measuring different isotopes can provide new information about palaeoenvironments. Passey *et al.* [66] explores the use of the ^17^O anomaly of natural waters that occurs owing to evaporation. Evaporated waters have a much lower Δ^17^O value than meteoric water, and in animals, the Δ^17^O relates to the intake of evaporated water [66]. Additionally, Δ^17^O is partially controlled by the O_2_ of the atmosphere; being able to measure it in fossil eggshells could give unprecedented information about the atmosphere and global primary productivity in deep time. Measurements of Δ^17^O in both modern bird eggshell and fossil dinosaur eggshell show that the recorded Δ^17^O value in biogenic materials strongly indicates the evaporated nature of water consumed by the animals, and once the systematics of the isotope system are better understood in modern eggshells, it will be a valuable palaeoenvironmental proxy for reconstructing the parent *δ*^18^O values of meteoric water in a locality [66].

## 4. Palaeobiological indicators

### 4.1. DNA and ancient DNA

Bird eggs and eggshells can be used as a substrate for non-invasive DNA analysis. Rare or reclusive birds can leave behind nests with egg material that provides a substrate for DNA analysis that is easier than handling and manipulating birds for a blood sample [67]. DNA can be obtained either from the eggshell membrane [68–71] or the hard portion of the eggshell that has been crushed or swabbed [67,72,73]. The location the DNA is taken from the eggshell determines which individual it is from—the parent or the offspring. DNA from the outside of the shell or the shell itself will be that of the parent that incubated or sat on the egg while the interior portion of the shell has DNA from the offspring [69].

The ability to sequence DNA from both modern and fossil bird eggshell can be used to taxonomically identify bird eggs in a variety of important cases, such as in wildlife forensics. The illegal pet trade is a multi-billion dollar business and exotic birds such as parrots and cockatoos are often trafficked for this purpose, and in Coghlan *et al.* [74] they were able to identify 52% of unknown eggs to species level and the ones that could not be identified to species level were identified to genus level. Eggshell DNA can help identification of unknown species in both forensic situations and also in museums, increasing the use of collections that may not be properly labelled.

The first instance of aDNA from fossil eggshells was published in Oskam *et al.* [75], where eggshell fragments yielded identifiable aDNA from archaeological sites up to 19 000 years old. DNA from extinct and extant bird genera such as *Dromaius*, *Tyto*, *Mullerornis*, *Genyornis*, *Dinornis* and *Aepyornis* were all sequenced from eggshell indicating the promising potential for long-term DNA preservation even in warm environments such as Australia and Madagascar. This study also uncovered where in the shell DNA is preserved by using confocal microscopy; the DNA is distributed evenly around the shell but may be concentrated more around the mammillary cones [75]. Experiments with quantitative polymerase chain reaction reveal that the bacterial contamination is 125 times less in eggshell than in bone, making it an ideal substrate for aDNA analysis. The DNA in the organic matrix contained within the eggshells is protected from environmental degradation by the crystalline matrix, making it more viable than porous bone.

In one of the other original studies of eggshell aDNA, Huynen *et al.* [76] were looking for DNA on both the inner and outer eggshell surfaces to not only figure out which species unknown eggshells were from, but to reveal which parent brooded on the egg during incubation. The detection of male DNA on the outside of 400–700 year old moa (*Dinornis*) eggshells from New Zealand could mean that male moa incubated eggs, which is similar to extant ratite birds [76]. Not only can aDNA be used to identify and sex the eggs, but it can also be used to detect haplotypes that reveal the structure of ancient populations of extinct moa. It was noted that there were in fact two distinct lineages of moa sampled in this study, opening the door for deep time population genetics using bird eggshells [76].

Subsequent studies using aDNA have been used for identifying not only what species of birds lived at archaeological sites, but the number of individuals. Oskam *et al.* [77] obtained both mitochondrial and nuclear DNA from eggshell fragments in a late thirteenth century oven at Wairau Bar in New Zealand. They were able to identify three genera of bird (*Emeus*, *Euryapteryx*, *Dinornis*) and also determine there were a minimum of 31 individual birds. Using more advanced sequencing methods, Allentoft *et al.* [78] used high-throughput sequencing technology to sequence microsatellites from aDNA in moa eggshells, allowing for population genetic studies in an extinct community. Not only is this method helpful for looking at palaeodiversity of birds at a site, but helps reveal the historical egg use by humans at an archaeological site.

Rapidly advancing genomic sequencing technology has recently been applied to eggshells of moa, allowing for a reconstruction of the phylogenetic history of palaeognaths and ratite birds where it was not before possible [79]. Previously, aDNA obtained from eggshells was mostly short fragments, but Grealy *et al.* [79] were able to use next-generation sequencing to obtain an entire mitochondrial genome and 12 500 base pairs of nuclear DNA from *Aepyornis* eggshell dated 1100 years BP. Using these DNA and phylogenetic dating methods, they were able to determine for the first time that the larger group of birds that ratites belong to, palaeognaths, underwent an evolutionary radiation between 69 and 52 Ma. Developments in aDNA sequencing will continue to revolutionize the uses of fossil material like eggshells, bringing new insights to deep-time radiations in the avian family tree.

### 4.2. Nest type and behaviour

The inherent morphological structure of eggshell contains signatures of nesting behaviour. The hard eggshell provides resistance to the diffusion of water, oxygen and carbon dioxide, but there must be some exchange with the environment for the embryo to survive. The orientation, density and number of eggshell pores that are used in gas exchange between the inside and outside of the egg can be used as a proxy for nesting behaviour and physiology. Experimental research shows gas conductance of an egg has a close relationship to the environment of incubation, indicating how exposed the egg is to the environment. This research, first pioneered in the 1970s, illustrated a correlation between nest structure and eggshell porosity in extant birds and developed an equation to be used to calculate a gas conductance value [80,81]. This idea was subsequently applied to inferring dinosaur nest physiology in Seymour [82]. The gas conductance is typically measured as water vapour conductance and denoted as $G_{{\text{H}}_{2}\text{O}}$ with units of mg H_2_O day^−1^ Torr^−1^.

Using water vapour conductance measurements as a proxy for nest type is based on a straightforward calculation born out by studies of modern eggshells. When eggs are buried, the humidity surrounding the egg is high, and the water vapour conductance values of the eggshells are also high [82,83]. Alternatively, eggs that are exposed to open air have reduced water vapour conductance to limit rapid diffusion of gas from inside the egg to the atmosphere. Seymour [82] used this basic principle to determine what type of nests dinosaurs made by measuring the number of pores and their density on enlarged photos of dinosaur eggshell surfaces and subsequently calculating a water vapour conductance value. The eggs from France and Mongolia's Gobi Desert both had significantly higher $G_{{\text{H}}_{2}\text{O}}$ than modern birds that mostly have open-air exposed nests, indicating adaptations to a low O_2_ environment such as a buried nest. This research was the first indication that non-avian dinosaurs buried their eggs in vegetation or covered them with sediment.

Additional research correlating nesting environment to water vapour conductance measured from shell morphology has strengthened the relationship and made the proxy even stronger for use in fossil studies [84,85]. For example, megapode birds that are unique for building nest mounds have higher $G_{{\text{H}}_{2}\text{O}}$ than other birds with open nests [86]. Gecko eggs that are incubated in open-air nests have low water vapour conductance [87] showing that this gas conductance and nest type correlations applies to both crown reptiles and birds. Although, it has been noted there have been two different methods used to measure water vapour conductance. Experimentally measured $G_{{\text{H}}_{2}\text{O}}$ values from modern birds have been compared to ones determined via pore geometry, so care must be taken when calculating nest type in extinct animals when the conductance values are determined through two different methods [88].

The discovery of a plethora of dinosaur eggs around the world, some of which are identifiable to species level because they have been found with embryos of parent dinosaurs, has led to water vapour conductance measurements being made on a large sampling of non-avian dinosaurs. Studies on non-avian dinosaur eggs from France, Mongolia, Romania, Spain and Argentina indicate similar nesting ecologies for sauropods, ornithischians and some theropods; the overwhelming consensus is that most of these non-avian dinosaur buried their eggs due to their high $G_{{\text{H}}_{2}\text{O}}$ values when compared to $G_{{\text{H}}_{2}\text{O}}$ of similarly sized bird eggs [84,85,89–95]. For example, oviraptorid eggs have a $G_{{\text{H}}_{2}\text{O}}$ that is 2.1 times higher than that expected from a similarly sized bird egg while an allosaurid egg has a $G_{{\text{H}}_{2}\text{O}}$ 7.0 times higher than expected [85].

The transition from buried nests to open nests probably occurred before or during the transition from non-avian dinosaurs to crown birds [96], so looking at non-avian dinosaur eggs may help pinpoint when this change in physiology and behaviour occurred. Some finer differentiation in non-avian dinosaur nesting environment has been found as eggs that have been attributed to sauropods do show differences in nest type between localities: for example a diagnosable titanosaur eggshell from Auca Mahuevo, Argentina has a lower $G_{{\text{H}}_{2}\text{O}}$ value than another eggshell of the same type from Spain, indicating that some species of sauropod did have a more open burial during nesting, although this study is based off of an extremely small sample size of eggshells [97]. Varricchio *et al.* [98] examined the porosity and water vapour conductance of *Troodon formosus* eggs, which are phylogenetically more similar to birds than some more basal non-avian dinosaurs. In that study and Deeming [85], *Troodon* has one of the lowest $G_{{\text{H}}_{2}\text{O}}$ values out of all non-avian dinosaurs when compared to modern birds, indicating they could have been slightly different than other dinosaurs or were just on the lower end of the typical theropod range for conductance values. Additional exploration of dinosaur eggshell gas conductance might lead to more specific inferences about transitions in nest type and behaviour between dinosaurs and birds.

The gas conductance of bird and reptile eggshells has been calculated, albeit less frequently than in non-avian dinosaurs. During the Palaeocene–Eocene Thermal Maximum the climate changed drastically and potentially impacted the physiology of land vertebrates at the time; a study by Donaire & Lopez-Martinez [99] looked at ratite bird eggshell from an unknown species at a locality in Spain to determine the water vapour conductance. What they found was an extremely reduced porosity when compared to modern birds and theropod dinosaurs. The $G_{{\text{H}}_{2}\text{O}}$ of the ratite was eight times lower than that of recent birds, indicating a nesting environment that was extremely dry and probably open to the air. The water vapour conductance of fossil reptiles has also been examined in turtles after the discovery of a Pleistocene nest of *Meiolania* turtle eggs from Lord Howe Island in Australia [100]. Modern turtles lay their eggs in a variety of settings, either on the surface of the ground or in holes/mounds. This is the first gas conductance analysis of a fossil turtle egg and shows that they have a $G_{{\text{H}}_{2}\text{O}}$ value 10 times higher than a bird egg of a similar size. This is comparatively similar to water vapour conductance of modern tortoises like *Kinixys erosa* that deposit their eggs in an excavated hole, so it is predicted *Meiolania* did the same [100]. There is further analysis needed of water vapour conductance of modern and fossil eggshells, especially in little studied groups like reptiles and modern birds, as nesting habits during periods of warming and/or cooling environments could show how animal physiology and behaviour is directly impacted by climate change.

Another indicator of nesting type is egg colour, as it can indicate the importance of camouflage or post-mating signalling [101]. Enhanced methods of chemical investigations of fossil eggs using chromatography can detect organic compounds that are responsible for egg colour in Late Cretaceous oviraptorid dinosaur eggshells. Wiemann *et al.* [101] found for the first time the presence of the organic compounds protoporphyrin and biliverdin, indicating the origin of blue-green eggs can be extended to non-avian dinosaurs, outside of crown birds. Modern crown birds with coloured eggs often have open nests [102], so the blue-green eggs of the oviraptorids provide valuable ecological information about these dinosaurs and their environment. Going forward, more detailed examination of egg colour in fossil shells from extinct species can broaden our knowledge of palaeoecology and behaviour not otherwise attainable from the fossil record.

### 4.3. Body temperature

Palaeontologists have long tried to estimate what the average body temperature of dinosaurs was, as their status as endotherms or ectotherms has been long debated [28]. As previously mentioned, stable isotope analysis usually involves examining a ratio of heavy to light isotopes, but a different kind of isotope geochemistry called clumped isotope geochemistry instead measures two or more heavy isotopes in the same molecule [103]. The number of heavy isotope CO_2_ ‘clumps’, known as the Δ_47_ value, measures CO_2_ with mass-47 (^13^C^18^O^16^O), which is correlated with the temperature of mineral formation [28]. Because eggs are formed within the oviduct of egg-laying animals, the temperature of mineral formation should reflect the body temperature of the ovulating female dinosaurs.

Clumped isotope measurements were made on the eggshells of extant birds and reptiles and the body temperature derived from *Δ*_47_ was found to closely match known body temperatures in the same animals [28]. The validation of this method in modern eggshells reveals its applicability for fossil dinosaur and avian eggshells. Well-preserved oviraptorid type eggshells from Mongolia's Gobi Desert yielded an estimated body temperature of 31.9 ± 2.9°C and a similarly preserved eggshell from an Argentine titanosaur has an estimated body temperature of 37.6 ± 1.9°C [28]. These body temperature estimates derived from *Δ*_47_ classify the oviraptor as intermediate between endotherms and ectotherms because its measured temperature is lower than most extant endotherms. This led the authors to conclude that dinosaurs may have possessed variable thermoregulation and not have belonged to either end-member thermoregulatory group [28]. Clumped isotope techniques on eggshells can be of critical use to test hypotheses of dinosaur and bird evolutionary physiology and the continued development of advanced isotope techniques will help increase the use of eggshells in both the modern and fossil record.

Traditional stable isotope methods can also be used to determine the incubation temperature of eggs laid by dinosaurs using the *δ*^18^O value of the eggshell carbonate and the *δ*^18^O value of the bone phosphate from embryos preserved inside the eggs [104]. The *δ*^18^O value of the eggshell carbonate reflects the oxygen isotope composition of egg water fluid; the bones of the embryo precipitate from the egg water. Because the isotope fractionation between the bone phosphate and egg water is temperature dependent, a time-dependent model can reveal the incubation temperature of the egg. Amiot *et al.* [104] used the measured isotope values of eggshell and embryo bones of seven different oviraptor specimens to determine the range of incubation temperature was between 35 and 40°C. Both methods, clumped isotope and creating a time-dependent model based on *δ*^18^O measurements, produce similar estimations for dinosaur body temperature, although the method described in Amiot *et al.* [104] necessitates the presence of both an embryo and an eggshell from the same individual, which is not commonly discovered.

## 5. Future directions

The variety of studies detailed above illustrates how useful eggs and egg products can be as a proxy for environment, diet and ecology in modern and deep time. Despite how useful eggs and eggshells can be there are still a number of areas that have not been explored. There remains little to no work done on the stable isotope geochemistry or microstructure of fossil and extant turtle, squamate or monotreme eggs. This is a major area of research that could allow for the use of more eggs in environmental and ecological study. Additionally, it is clear more time needs to be focused on experiments with controlled feeding with captive birds and reptiles so the geochemical examinations of the eggshell can be used for determining ecology and biological parameters like body temperature. The development of new advanced isotope methods like clumped isotopes and Δ^17^O shows that the continuous innovation of geochemical methods for carbonates can be tested on eggs to create new palaeoenvironmental proxies. Owing to the availability of eggs and egg products in museum collections, modern ecosystems and fossil deposits, any future research on this subject will allow us a better and fuller understanding of the biology and ecology of egg-laying vertebrates.

## Data accessibility

There are no data associated with this paper.
